# Synthetic-biology approach for plant lignocellulose engineering

**DOI:** 10.5511/plantbiotechnology.24.0630a

**Published:** 2024-09-25

**Authors:** Kouki Yoshida, Shingo Sakamoto, Nobutaka Mitsuda

**Affiliations:** 1 Technology Center, Taisei Corporation, Yokohama, Kanagawa 245-0051, Japan; 2 Bioproduction Research Institute, National Institute of Advanced Industrial Science and Technology (AIST), Tsukuba, Ibaraki 305-8566, Japan; 3 Global Zero Emission Research Center, National Institute of Advanced Industrial Science and Technology (AIST), Tsukuba, Ibaraki 305-8566, Japan; 4 Bioproduction Research Institute, National Institute of Advanced Industrial Science and Technology (AIST), Sapporo, Hokkaido 062-8517, Japan

**Keywords:** biomass, cell wall, hemicellulose, lignin, lignocellulose engineering

## Abstract

Plant biomass is an abundant, renewable resource that offers multiple advantages for the production of green chemicals and recombinant proteins. However, the adoption of plant-based systems by industry is hindered because mammalian and other cell cultures are well-established and better characterized in an industrial setting, and thus it is difficult for plant-based processes to gain a foothold in the marketplace. Therefore, additional benefits of plant-based systems may be essential to tip the balance in favor of sustainable plant-derived products. A crucial factor in biomass valorization is to design mid- to high-value co-products that can be derived cost-effectively from the residual lignocellulose (LC). However, the utility of LC remains limited because LCs are, in general, too recalcitrant for industries to utilize their components (lignin, cellulose, and hemicelluloses). To overcome this issue, in planta engineering to reduce LC recalcitrance has been ongoing in recent decades, with essential input from synthetic biology owing to the complexity of LC pathways and the massive number of genes involved. In this review, we describe recent advances in LC manipulation and eight strategies for redesigning the pathways for lignin and structural glycans to reduce LC recalcitrance while mitigating against the growth penalty associated with yield loss.

## Introduction

Tens of billions of tonnes of terrestrial plant biomass are produced annually of which lignocellulose (LC) represents the primary source. Cell walls, which are composed predominantly of LC, constitute more than half of plant dry biomass (Pauly and Keegstra 2008). However, the utility of LC remains limited because of its properties and complexity. More specifically, LCs are recalcitrant to enzymatic conversion despite a variety of bioeconomic, political, and social demands for industrial use of their individual components (lignin, cellulose, and hemicelluloses). To overcome this barrier, the following two approaches for weakening plant cell walls have been studied in recent decades: (1) thermochemical or enzymatic treatments of LC, and (2) in planta LC engineering. The latter is based on plant biotechnological techniques, including synthetic biology, such as genome editing, multigene stacking, and generation of novel enzyme activity (Mortimer 2019). The LC synthesis pathways are directly or indirectly linked to the production of diverse, high-value secondary metabolites (green chemicals) through the phenylpropanoid biosynthesis pathway (PPP) for caffeoylquinic acids (Alcázar Magaña et al. 2021), the shikimate pathway (SKP) for antimicrobial agents, including manzamine alkaloids (Almeida et al. 2024), and the *S*-adenosylmethionine (SAM or AdoMet) pathway for methylation of many intermediates for green chemicals. Thus, the manipulation of LC synthesis pathways affects green chemical production.

The LC synthesis pathways are complex and involve a substantial number of genes. For example, the majority of genome-wide association studies on grass species indicate that the effects of individual single-nucleotide polymorphisms associated with cell wall components are minimal and explain only a small proportion of the total phenotypic variance (López-Malvar et al. 2019), reflecting the complexity of the genetic basis of biomass recalcitrance (De Lorenzo et al. 2019). Thus, cell wall components and the associated phenotypes of graminaceous crops might be controlled by many minor-effect genes, as concluded by Li et al. (2016). This implies that cell wall engineering in graminaceous crops requires the step-wise introduction of gene clusters to improve LC properties. From a reverse genetic perspective, the Arabidopsis genome contains 1302 genes encoding carbohydrate-active enzymes and carbohydrate-binding modules (CBMs) based on information in the CAZY database (http://www.cazy.org/e1.html (Accessed Aug 24, 2024)). This number includes genes that code for more than 450 glycosyltransferases (GTs) classified into more than 40 distinct GT families (Lao et al. 2014). The development of powerful tools to simultaneously edit or activate multiple genes (Pan et al. 2022) would accelerate the bioengineering of cell walls in seed plants. Synthetic biology has the potential to revolutionize the production of manipulated plant LCs under conventional cropping and could be applied to create efficient cell lines that produce high-value and medicinal glycans in indoor environments (Voiniciuc 2023). In this review, we briefly describe recent advances in plant cell wall biology and outline several strategies, partially assisted by plant synthetic biology, for redesigning the lignin and cell wall glycan pathways to reduce LC recalcitrance while mitigating against the growth penalty resulting from yield loss.

## Structural basis for lignocellulose recalcitrance

Lignin is a complex polymer derived primarily from oxidative coupling of *p*-hydroxycinnamyl alcohols comprising three canonical monolignols (MLs), namely, *p*-coumaryl, coniferyl, and sinapyl alcohols. In the lignin polymer, these MLs form *p*-hydroxyphenyl (H), guaiacyl (G), and syringyl (S) units, respectively. Lignin units are interconnected primarily through β-*O*-4 ether bonds together with very minor α-*O*-4 ether bonds, both of which are susceptible to chemical or biochemical cleavages, and smaller amounts of so-called “condensed” C-C (β–5, β–β, and 5–5) and biphenyl ether (4-*O*-5, and 5-*O*-4) bonds that are resistant to chemical degradation (Grabber et al. 2004; Ralph et al. 2019). Lignins have been generally characterized in three major groups of plant based on the composition ratio of the MLs and non-canonical monomers: G-lignin (S : G 0 : 100) in softwoods (gymnosperms), G-S lignin (S : G 65 : 35) in hardwoods (angiosperms), and G-S lignin (S : G 68 : 32) with ferulic acids (FA) or tricin unit in grasses (Gramineae), while the level of H-units is rarely above 5% as can be determined by NMR or more diagnostic degradative analytical methods regardless of plant species (Ralph et al. 2019).

Lignin has abundant electrostatic interactions with the polar motifs of xylan (Kang et al. 2019) and is linked to the primary hydroxyl group of a sugar residue in glucomannan through an α ether bond (Nishimura et al. 2018). In lignified tissues of grasses, FA esterified to arabinoxylan (AX) can also be etherified to lignin polymer units via oxidative radical coupling, forming covalent linkages between AX and lignin (Hatfield et al. 2016), which contributes to biomass recalcitrance. Furthermore, lignin binds preferentially to the hydrophobic surface of cellulose, which is also the preferred binding site for the fungal cellulase TrCel7A, thereby amplifying the competitive inhibitory effect (Vermaas et al. 2015). In addition, lignin forms specific interactions with three tyrosine (Tyr) residues on the CBM of TrCel7A, representing a second mechanism for competitive inhibition. Elimination of the Tyr–lignin interactions of TrCel7A may be particularly difficult to engineer because mutations to the CBM that disrupt the interaction with lignin will likely also reduce the affinity of the CBM for cellulose. Engineering of lignin within plant biomass may be a preferable approach (Vermaas et al. 2015), possibly by enhancing the hydrophobicity such that it adopts a more compact state (Carmona et al. 2015) and presents a smaller interaction surface area. Therefore, lignin removal is a crucial step in biomass conversion to biofuels and green chemicals that motivates efforts to re-engineer lignin biosynthesis. To date, there is a rich history in basic research for lignin and polysaccharide bioengineering and there are excellent reviews on these subjects (Allen et al. 2021; Anders et al. 2023; Pedersen et al. 2023; Ralph et al. 2023; Umezawa et al. 2020; Ye and Zhong 2022).

## Five strategies for redesigning the lignin pathway

**Figure 1:**
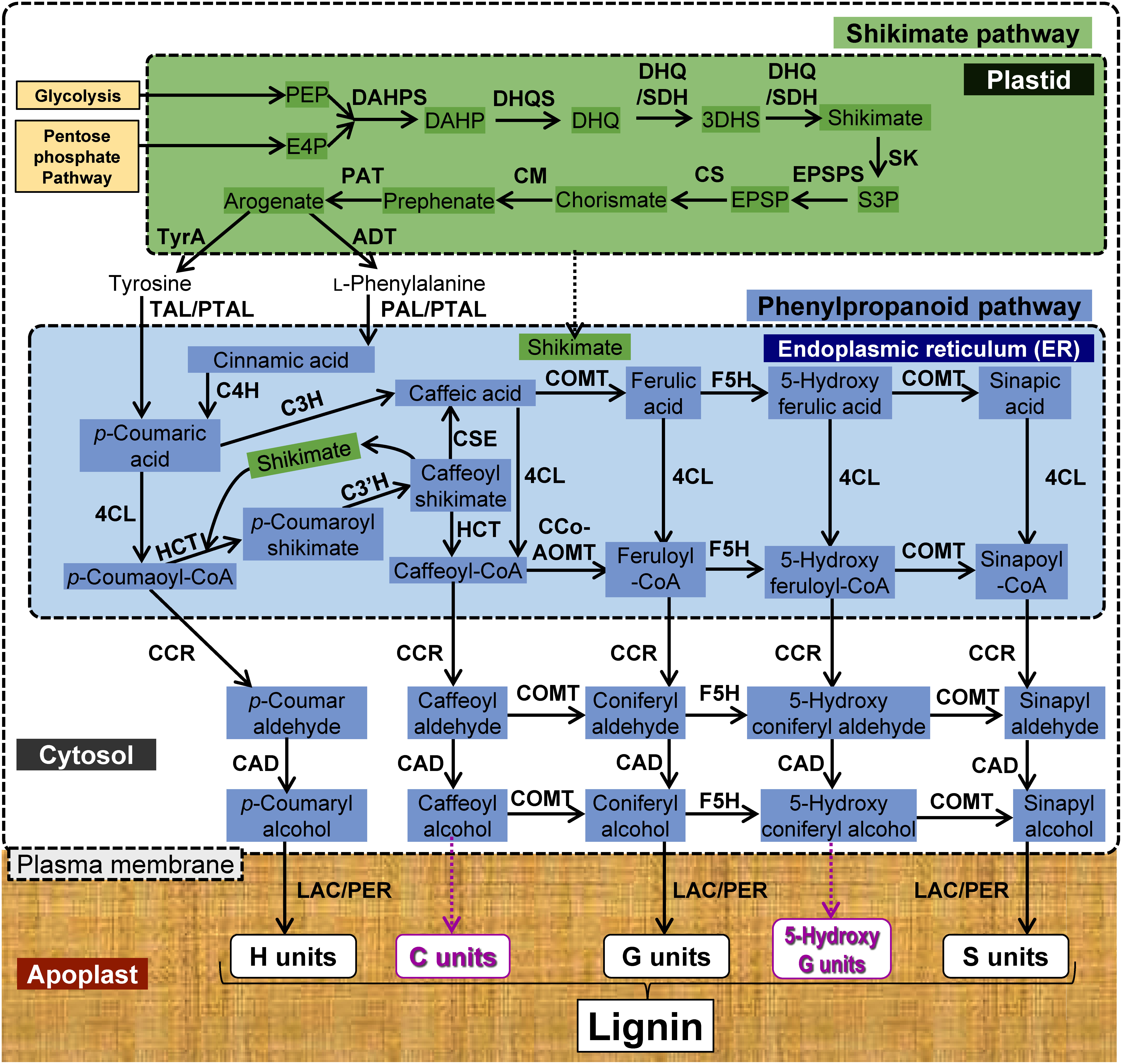
Figure 1. Schematic diagram of lignin biosynthesis pathway in plant cell. The magenta arrows and letters indicate unconventional steps and units of lignin after some genetic manipulations, respectively (F5H overexpression in the COMT mutant for 5-Hydroxy G units or down regulations of the methionine and folate cycles for C units, see also Figure 2). The shikimic pathway in plastids, phenylpropanoid pathway in cytosol and endoplasmic reticulum (ER) are described. Lignin units are H (hydroxyphenyl), G (guaiacyl) and S (syringyl). ADT, arogenate dehydratase; C3H, *p*-coumarate 3-hydroxylase; C3′H, *p*-coumaroyl-shikimate 3′-hydroxylase; C4H, cinnamate 4-hydroxylase; CAD, cinnamyl alcohol dehydrogenase; CCoAOMT, caffeoyl-CoA *O*-methyltransferase; CCR, cinnamoyl-CoA reductase; CM, chorismate mutase, COMT, caffeate/5-hydroxyferulate *O*-methyltransferase; CS, chorismate synthase; CSE, caffeoyl shikimate esterase; C units, catechyl units; DAHP, 3-deoxy-d-arabino-heptulosonate 7-phosphate; DAHPS, 3-deoxy-d-arabino-2-heptulosonate 7-phosphate synthase; DHQ, 3-dehydroquinate; 3DHS, 3-dehydroshikimate; DHQS, 3-dehydroquinate synthase; DHQ/SDH, 3-dehydroquinate dehydratase/shikimate 5-dehydrogenase; E4P, d-erythrose 4-phosphate; EPSP, 5-enolpyruvyl shikimate-3-phosphate, EPSPS, 5-enolpyruvylshikimate 3-phosphate synthase; F5H, ferulate 5-hydroxylase or coniferyl aldehyde 5-hydroxylase; HCT, *p*-hydroxycinnamoyl-CoA shikimate hydroxycinnamoyl transferase; LAC, laccase; PAL, phenylalanine ammonia-lyase; PAT, prephenate aminotransferase; PEP, phosphoenol pyruvate; PER, peroxidase; PTAL, a bifunctional phenylalanine and tyrosine ammonia-lyase from grass; TAL, tyrosine ammonia-lyase; TyrA, tyrosine arogenate dehydrogenase; S3P, shikimate-3-phosphate; SK, shikimate kinase.

The lignin biosynthesis pathway is localized in at least five compartments of plant cells: SKP in the chloroplast, PPP in the endoplasmic reticulum (ER) and cytoplasm, ML transport probably across the plasma membrane (PM), and MLs polymerization in the cell wall (Figure 1). To date, five major strategies to manipulate the lignin pathways have been applied.

### (1) Negative manipulation of lignin contents

In the initial phase of studies on lignin engineering, genes encoding enzymes involved in ML biosynthesis such as *CINNAMATE 4-HYDROXYLASE* (*C4H*) and *CINNAMOYL-CoA REDUCTASE* (*CCR*), were suppressed to manipulate the lignin content (Figure 1, Simmons et al. 2010). Strategies to decrease the total lignin content of plants have often been associated with undesirable yield losses, i.e., there is a growth penalty (Muro-Villanueva et al. 2019). The lignin-related growth penalty has been termed lignin modification-induced dwarfism (LMID) (Muro-Villanueva et al. 2019), implying that it is attributable directly to the modification of lignin as a structural requirement to support water transport. Several models for LMID have been proposed that the associated dwarfism is partially due to hyperaccumulation of pathway products, such as *cis*-cinnamic acid interfering with auxin transport as demonstrated in a T-DNA insertion mutant of *C4H* (El Houari et al. 2021), or activation of cell wall integrity surveillance mechanisms as observed in T-DNA insertion mutants of *CCR1* (*ccr1*, Gallego-Giraldo et al. 2020), through the release or perception of elicitor-active pectic glycans involving the receptor-like kinase *FERONIA* or wall-associated protein kinases (Liu et al. 2023). The latter case suggests that plant cell wall engineering will require approaches that bypass the endogenous pectin signaling pathways (Liu et al. 2023).

The LMID problem has been mitigated by vessel-specific complementation in lignin-deficient mutants, such as the *ccr1* mutant (Yang Y et al. 2022). The expression of *CCR1*, under the control of a synthetic promoter containing multiple secondary cell wall (SCW) NAC binding elements, in the *ccr1* mutant resulted in the mutant having restored biomass yield and a fourfold increase in total sugar yield by enzymatic saccharification compared with wild-type (WT) plants (De Meester et al. 2018). Subsequently, the inducible synthetic promoter was incorporated into a single DNA construct in which CRISPR-Cas9 gene editing is used to knockout the *CCR1* gene, while a modified version of the same gene with a mutation in the open reading frame to escape Cas9 digestion is expressed in the xylem (Yu et al. 2021). This toolkit provides a rapid and systematic approach for molecularly breeding lignin-reduced trees and crops in a single transformation and, in combination with tissue-specific promoters, represents a general strategy for conditioning loss-of-function traits associated with LMID. The approach may be transferable to plant species other than Arabidopsis where both transformation and gene editing are practicable and where xylem-specific promoters are optimized not only for *CCR* but also for other PPP genes (Yu et al. 2021).

### (2) Manipulation of the lignin composition

**Figure 2:**
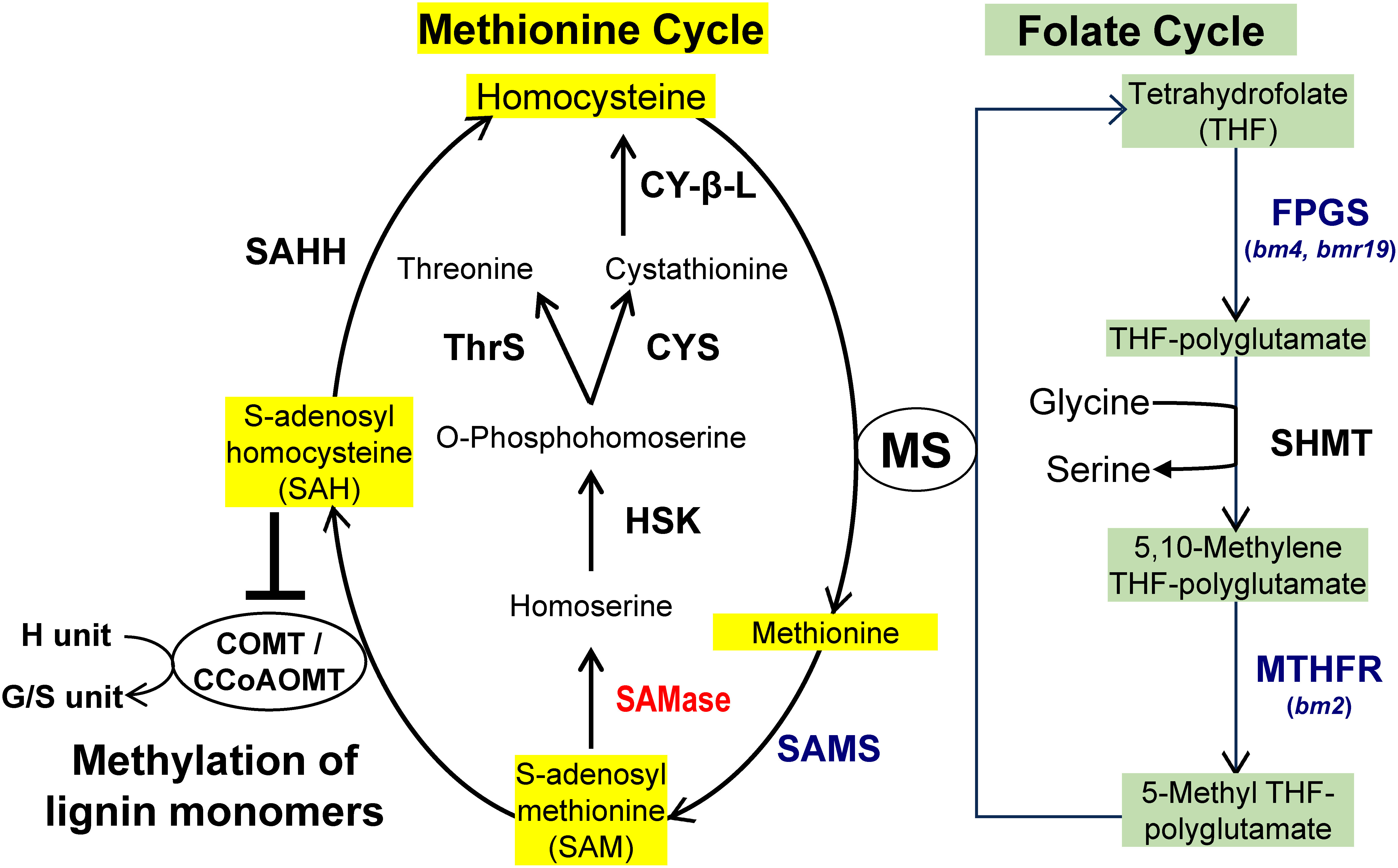
Figure 2. Interconnection between the lignin biosynthetic pathway and the methionine and folate cycles (Adapted from Liu and Eudes 2022 and Eudes et al. 2016b). Enzymes are: CCoAOMT, caffeoyl-CoA *O*-methyltransferase; COMT, caffeate/5-hydroxyferulate *O*-methyltransferase; CY-β-L, cystathionine β-lyase; CYS, cystathionine synthase; FPGS, folylpolyglutamate synthase encoded by maize brown midrib4 (*bm4*) gene or sorghum brown midrib19 (*bmr19*) gene; HSK, homoserine kinase; MS, methionine synthase; MTHFR, methylenetetrahydrofolate reductase encoded by maize brown midrib2 (*bm2*); SAHH, S-adenosylhomocysteine hydrolase; SAMase, S-adenosylmethionine hydrolase from coliphage T3 in red; SAMS, SAM synthase in blue, SHMT, serine hydroxymethyl transferase; ThrS, threonine synthase; SAH, S-adenosylhomocysteine; SAM, S-adenosylmethionine; THF, tetrahydrofolate. Red and Blue characters are genes which up regulated genes and down regulated, respectively.

*CAFFEOYL CoA-O-METHYLTRANSFERASE* (*CCoAOMT*, Figure 1) is involved in the first methylation step required for the production of G units and *CAFFEIC ACID O-METHYLTRANSFERASE* (*COMT*, Figure 1) performs the second methylation step needed for the synthesis of S units. Both enzymes consume SAM for transmethylation reactions (Figure 2). Modification of the intracellular pools of SAM has been proposed for alteration of lignin biosynthesis (Liu and Eudes 2022). Mutation of one Arabidopsis *SAM SYNTHETASE* gene (*AtSAMS3*) causes reduction in the SAM pools and lignin content (Figure 2, Shen et al. 2002). RNAi-directed downregulation of a homolog (*PvSAMS*) in switchgrass (*Panicum virgatum* L.) reduces the contents of SAM, G-lignin and S-lignin. These changes result in brownish stems associated with reduced lignin content and improved cell wall digestibility (Li et al. 2022). Mutations in *METHYLENE TETRAHYDROFOLATE REDUCTASE* (*MTHFR*) or *FOLYLPOLYGLUTAMATE SYNTHASE* (*FPGS*, Figure 2) involved in the synthesis of 5-methyltetrahydrofolate, which is used as a methyl donor by methionine synthase for the production of methionine from homocysteine, cause reduction in lignin contents in maize and Arabidopsis (Li et al. 2015; Srivastava et al. 2015; Tang et al. 2014). Curiously, the Arabidopsis *fpgs* mutant with short primary roots and root hairs does not show any change in aboveground biomass yield. The targeted expression of *SAM HYDROLASE* (*SAMase*, Figure 2) from Enterobacteria phage T3 in Arabidopsis tissues synthesizing SCWs reduces the SAM pool and impacts the lignin content (decreased by 27–31%) and composition (relative enrichment of non-methylated H units and reduced content of di-methylated S units; Eudes et al. 2016b). Biomass of the transgenic lines, compared with that of the WT, was enriched in glucose (Glc) content (by 9–13%) and the degree of glucuronoxylan methylation was reduced (by 72%). In addition, the transgenic lines were fertile and showed no discernible growth defects nor a decrease in total stem biomass, albeit with a 12–20% reduction in height of the main stem. These modifications resulted in an increase in sugar yield by enzymatic biomass saccharification compared with that of WT plants (by 26–29%). Considering that the transgenic plants showed no significant attenuation in biomass yield, and that manipulation of the SAM pool could be spatiotemporally optimized, this strategy provides a viable option for improvement of LC biomass feedstock.

The demethylated product of SAM, *S*-adenosylhomocysteine (SAH, Figure 2), may be an important factor for manipulation of the lignin pathway because SAH is a strong inhibitor of CCoAOMT and COMT activities (Liu and Eudes 2022). Moreover, the ratio of SAM : SAH but not the SAM concentration critically determines the catalytic efficiency of these enzymes and the transmethylation reactions in ML biosynthesis (Wu et al. 2018). Genetic analysis of the maize *brown midrib 2* (*bm2*) mutant has revealed that MTHFR (Figure 2), which catalyzes the conversion of 5,10-methylene-tetrahydrofolate to 5-methyl-tetrahydrofolate, is downregulated and affects the content of SAH rather than SAM. Consequently, the *O*-methylation of lignin monomers by CCoAOMT and COMT is impaired, which results in substantial accumulation of novel phenolics (caffeoyl alcohol glucoside derivatives and a caffeoylquinic acid) and unusual catecyl (C) lignin derived from the polymerization of caffeoyl alcohol (Figure 1) in the mutant (687.7% higher than in the WT). Although S units are not impacted, the altered lignin composition leads to a significant improvement in the cell wall saccharification efficiency (a relative increase of 57.8%). Further investigation is needed to determine whether the C units are incorporated into the canonical G-S lignin or synthesized in homopolymers, as has been observed in the seed coats of *Vanilla planifolia* and seeds of certain Cactaceae species (Chen et al. 2012). The predominant benzodioxane linkages in C-lignin as well as its linear polymeric structure may be advantageous for diverse lignin-based biomaterials (Berstis et al. 2016) and for the production of catechols as drug precursors (Li et al. 2023). In addition, the near-exclusive benzodioxane linkage between monomers indirectly prevents benzyl ether and ester crosslinking of hemicellulosic alcohol or acid groups with C units (Vanholme et al. 2012). Therefore, accumulation of C-lignin (and benzodioxane-type lignins in general) in bioenergy crops has been proposed to reduce cell-wall crosslinking between lignin and glycans (Grabber et al. 2019).

**Figure 3:**
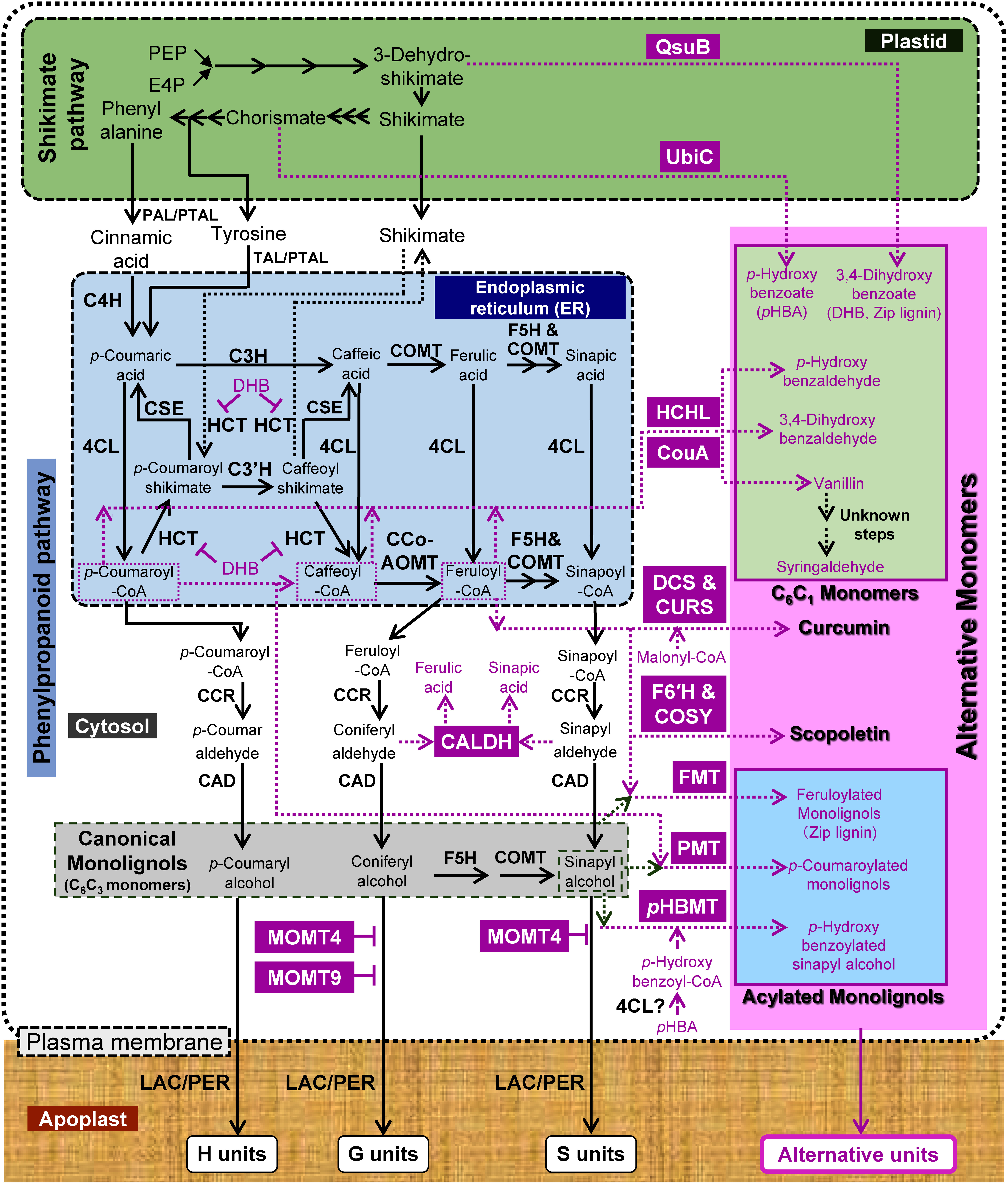
Figure 3. Manipulation of the lignin biosynthetic pathway based on studies from transgenic plants involving of heterologous expression of bacterial, plant and newly designed enzymes. Canonical monolignols and alternative monomers are biosynthesized from the shikimic pathway and phenylpropanoid pathway. Black character and arrows indicate metabolites and enzymes in the pathway for canonical monolignols biosynthesis. Magenta character and arrows indicates metabolites and enzyme for alternative monomer biosynthesis. Abbreviations are as follows, E4P, erythrose 4-phosphate; PEP, phosphoenolpyruvate; TAL, tyrosine ammonia-lyase; LAC, laccase; PER, peroxidase (See also Figure 1). Enzyme in black indicate the conventional steps in lignin pathway. Enzymes in magenta indicate the up-regulated genes. CALDH, coniferyl aldehyde dehydrogenase; CouA, hydroxycinnamoyl-CoA hydratase/lyase, DCS:CURS2, diketide-CoA synthase and curcumin synthase2; F6′H:COSY, feruloyl-CoA 6′-hydroxylase 1 and coumarin synthase; FMT, feruloyl-CoA monolignol transferase; HCHL, hydroxycinnamoyl-coa hydratase-lyase; MOMT4 and MOMT9, monolignol *O*-methyl transferase; pHBMT, *p*-hydroxybenzoyl-CoA monolignol transferase; PMT, *p*-coumaroyl-CoA monolignol transferase; QsuB, 3-dehydroshikimate dehydratase; UbiC, chorismate pyruvate lyase. The metabolic inhibition of HCT by QsuB-synthesized 3,4-dihydroxybenzoate (DHB) is also indicated.

The shuttle routes of “coniferyl aldehydes ↔ FA” and/or “sinapyl aldehydes ↔ sinapic acids” may interfere with the final step catalyzed by CINNAMYL ALCOHOL DEHYDROGENASES (CADs) in the PPP (Figures 1 and 3, Sakamoto et al. 2020). Microbial CONIFERYL ALDEHYDE DEHYDROGENASE (CALDH, Figure 3) of *Pseudomonas* sp. strain HR199 catalyzes the NAD^+^-dependent oxidation of coniferyl aldehyde to ferulic acid. In addition to coniferyl aldehyde, *trans*-cinnamaldehyde, sinapyl aldehyde, and benzaldehyde may be substrates of CALDH, with maximum percentages of approximately 97%, 77%, and 18% relative to coniferyl aldehyde, respectively (Achterholt et al. 1998). Arabidopsis lines expressing a CALDH gene, *calB*, under the control of *pAtC4H* produce less lignin but exhibit an increase in the lignin S/G ratio and improvement in saccharification efficiency (by 21%) after pretreatment without a severe growth penalty (Sakamoto et al. 2020). Initially, *calB* expression was expected to reduce coniferyl aldehyde and sinapyl aldehyde accumulation, and increase contents of the corresponding acids, while the expression levels of probable acyltransferases for cell wall polymers in Arabidopsis remain unclear (Yu et al. 2009). However, unexpectedly, the amounts of cell wall-bound *p*-coumarate (*p*CA) and FA were lower than those of the WT, while the sinapic acid abundance was below the detection limit in the *calB* line and WT (Sakamoto et al. 2020). The authors speculated that this might reflect the significantly higher *K*_m_ value of calB for coniferyl aldehyde (334 µM) (Achterholt et al. 1998) compared with those of Arabidopsis CAD-4 (65 µM) and CAD-5 (35 µM) (Kim et al. 2004). Nevertheless, the monomeric composition of lignin was altered and the saccharification efficiency was significantly improved in the *calB* line, although the mechanism was unclear. Liu and Eudes (2022) speculated that, in these lines, reduction of the coniferyl aldehyde pools may cause the changes in lignin monomeric composition considering that coniferyl aldehyde is not only the precursor of coniferyl alcohol and G units, but is also a potential inhibitor of CAD-catalyzed reduction of sinapyl aldehyde to sinapyl alcohol (Wang et al. 2014). Metabolomic analysis may clarify the role of *calB* in regulation of the cytosolic PPP resulting in improvement in LC recalcitrance.

### (3) Incorporation of alternative monomers in lignin

In recent decades, extensive research on the lignin pathway in mutants and transgenic plants has revealed that several phenolic compounds other than canonical monomers (H, G and S), derived from the PPP or the biosynthetic pathways of other polyphenols (e.g., flavonoids), may behave as lignin monomers in many plants and are integrally incorporated into the lignin structure (del Río et al. 2022). Examples of such compounds are phenolic compounds derived from incomplete biosynthesis of MLs in *CAD*-deficient mutants and *CONIFERYL ALDEHYDE 5-HYDROXYLASE* (*FERULATE 5-HYDROXYLASE*, F5H)-overexpressing transgenic lines of c*omt1* mutants (Figure 1, Weng et al. 2010), ML ester conjugates with various esters, such as *p*CA and FA, catalyzed by acyltransferases, curcumin, and the recently discovered coumarin scopoletin might be considered a lignin monomer (Figure 3). These alternative monomers have been a focus of genetic engineering to produce lignin with enhanced lability.

#### a) Scopoletin

Scopoletin is a coumarin that is naturally present in some plants. It is produced by 6-hydroxylation of ferulate by *FERULOYL-CoA 6′-HYDROXYLASE 1* (*F6′H1*), and subsequent ring closure after *cis*–*trans* isomerization by *COUMARIN SYNTHASE* (*COSY*) (Figure 3, Vanholme et al. 2019). It has been predicted that incorporation of scopoletin via its *O*-4 position into lignin may generate β-aryl ethers with a conjugated carbonyl function. The carbonyl functionality is believed to significantly decrease the hydrolysis temperature required for the cleavage of aryl ethers in an alkaline environment. Furthermore, alkaline hydrolysis of the ester functionality of scopoletin results in the release of a carboxylic acid and an additional free phenolic group. This enhances the hydrophilicity and alkaline solubility of the pretreated polymer, which promotes lignin extraction in aqueous solvents (Hoengenaert et al. 2022). Scopoletin has been incorporated into lignin in Arabidopsis expressing *F6′H1* under the control of the *Populus sieboldii*×*Populus grandidentata* *C4H* promoter (Sakamoto et al. 2020). In addition, scopoletin has been incorporated into the lignin polymer with greater efficiency in Arabidopsis through simultaneous expression of *F6′H1* and *COSY* with a bicistronic construct linked by a T2A sequence under the control of the SCW-specific *CELLULOSE SYNTHASE 4* (*CesA4*) promoter (Hoengenaert et al. 2022). Approximately 3.3% of the lignin units in the transgenic lines were derived from scopoletin, thereby exceeding the content of canonical H units without adversely affecting plant growth. The saccharification efficiency of alkali-pretreated scopoletin-overproducing lines was 40% higher than that of the WT.

#### b) Curcumin

The polyketide curcumin, a potentially novel ML, was overproduced in Arabidopsis by rerouting of feruloyl-CoA under heterologous co-expression of *DIKETIDE-CoA SYNTHASE* (*DCS*) and *CURCUMIN SYNTHASE 2* (*CURS2*) from turmeric (Figure 3, *Curcuma longa*; Oyarce et al. 2019). Curcumin has been synthesized and incorporated into lignified cell walls of Arabidopsis. In addition, the transgenic Arabidopsis plants did not suffer yield penalties and the saccharification yield after alkaline pretreatment was increased. These improvements may reflect the presence in lignin of alkali-labile β-diketone structures arising from the incorporation of curcumin (Liu and Eudes 2022). Poplar expressing *DCS* and *CURS2* under the control of the SCW *CELLULOSE SYNTHASE A8-B* promoter (*ProCesA8-B*) from *P. trichocarpa* has been generated (De Meester et al. 2022). However, in contrast to Arabidopsis, the latter lignin-engineering strategy results in increased lignification, significantly altered lignin composition, growth disruption, and shoot-tip necrosis in transgenic poplar. These phenotypic differences illustrate the importance of translational research in crops.

#### c) *p*-Coumaroylation of monolignols

Lignin from several plants can be acylated at its side chains with various groups, including *p*CA and *p*-hydroxybenzoate (*p*HBA). *p*CA is distributed in diverse herbaceous angiosperms, including grasses, kenaf (*Hibiscus cannabinus*) (del Río et al. 2008), and mulberry (*Morus* spp.; Hellinger et al. 2023). *p*HBA is present in lignin of palms (Lu et al. 2015) and poplars (de Vries et al. 2022; Zhao et al. 2021). The upgrading of aromatic compounds, such as *p*CA, into more valuable bioproducts represents an option for establishment of economically sustainable biorefineries (Liu and Eudes 2022). Ester-linked groups are usually more frequent in lignin S units than in G units in land plants.

*p*-Coumaroylation involves acyltransferases that catalyze the formation of ML conjugates using CoA-activated acyl groups as donors and MLs as acceptors. Acyltransferases responsible for the formation of ML *p*CA conjugates (*p*-*COUMAROYL-CoA:MONOLIGNOL TRANSFERASE*, *PMT*, Figure 3) have been identified in several grass species, including *Brachypodium distachyon* (*BdPMT1*, Petrik et al. 2014, *BdPMT2*, Sibout et al. 2016) and rice (*OsPMT1*, Withers et al. 2012, *OsPMT2*, Lam et al. 2024). Grass *PMT* has been exploited to engineer lignin in Arabidopsis and poplar without impacts on plant growth (Lapierre et al. 2021; Sibout et al. 2016; Smith et al. 2015). An advantage of this strategy is that increased *p*-coumaroylation of lignin favorably affects lignin structure: engineered highly *p*-coumaroylated lignins contain an increased proportion of terminal units with free phenolic groups, presumably a reduced degree of polymerization, and higher solubility in an alkaline solution, which improves subsequent enzymatic saccharification of biomass (Lapierre et al. 2021; Sibout et al. 2016). In contrast, heterologous expression of kenaf *PMT* in hybrid poplar led to the incorporation of *p*CA in lignin but no improvement in the saccharification potential of the wood (Mottiar et al. 2023b).

An additional benefit of increased *p*-coumaroylation is enrichment of lignin with *p*CA, which can potentially be easily cleaved by alkali pretreatment on account of the ester linkages, and recovered from lignin streams generated in biorefineries (Liu and Eudes 2022). A goal of target extraction titers is for one hydroxycinnamic acid (*p*CA or FA) >50 g kg^−1^ in biomass of genetically engineered maize cobs, at least five-times higher than observed titers for the impure *p*CA/FA product mixture from WT maize. Future plant-engineering efforts to enhance accumulation of one predominant type of hydroxycinnamate and suppress accumulation of the other type might be economically competitive for the petrochemical industry. In addition, a technical challenge for process engineering is to develop a viable procedure that requires more than 80% reduction in the isolation costs (Karlen et al. 2020).

#### d) Zip-lignin resulting from plant FMT or microbial *QsuB* activity

In contrast to ML-*p*CA conjugates, which are predominantly incorporated into lignin only via the ML moiety, ML-FA conjugates can be incorporated into lignin through the conjugated FA unit as well (Wilkerson et al. 2014). This enables the introduction of unique ester bonds into the lignin backbone that are considerably more reactive than the dominant ether bonds and should readily “unzip” during chemical processing (Zhou et al. 2017). Such lignin is termed “Zip-lignin”. Screening of a large number of plant species revealed that ML-FAs were widely present in the lignin of many species, including WT poplar, albeit in low amounts (0.3 mg g^−1^ of acetyl bromide soluble lignin; Karlen et al. 2016). Initial research on incorporation of ML-FAs into lignin was performed by engineering poplar to express a gene that encodes a *FERULOYL-CoA MONOLIGNOL TRANSFERASE* (*AsFMT*, Figure 3) from *Angelica sinensis*, a dicotyledonous Chinese medicinal plant (Wilkerson et al. 2014). The AsFMT couples MLs with feruloyl-CoA to produce ML-FAs and homologues have been identified from grass species, such as the *OsFMT1* (*AT5*) gene (Karlen et al. 2016). Transgenic *AsFMT*-expressing poplar produced ML-FAs and used them in lignification (five- to seven-fold increase in ML-FAs from acetyl bromide soluble lignin compared with the amount in the WT), resulting in improved cell wall saccharification after mild base pretreatment and the absence of phenotypic growth abnormalities (Wilkerson et al. 2014). Subsequently, kraft pulping of Zip-lignin and WT hybrid poplar wood was performed in laboratory-scale reactors under conditions of varying severity by altering the duration, temperature, and chemical charge (Zhou et al. 2017). Statistical prediction models facilitated comparisons between pulping conditions that resulted in identical delignification. The Zip-lignin poplar needed milder cooking conditions and resulted in higher pulp yield (as much as 1.41% gain). Bleaching and physical properties were subsequently equivalent between the samples, with slight chemical savings realized in the Zip-lignin samples owing to enhanced delignification (Zhou et al. 2017).

In addition to plant-derived genes, the heterologous expression of genes from microorganisms is an alternative strategy for incorporation of novel lignin monomers in planta. In hybrid poplar expressing a *3-DEHYDROSHIKIMATE DEHYDRATASE* (*QsuB*, Figure 3) gene from the bacterium *Corynebacterium glutamicum* under the control of the *Arabidopsis C4H* promoter, the lignin content was decreased (by as much as 33% with *p*-hydroxyphenyl units comprising as much as 10% of the lignin) and biomass saccharification efficiency was increased (as much as 40% increase in Glc release), with no overall significant adverse effects on biomass yield (Unda et al. 2022). The *QsuB* gene has been targeted to plastids to convert 3-dehydroshikimate, the direct precursor of shikimate, into 3,4-dihydroxybenzoate (or protocatechuate, DHB) or 3,4-dihydroxybenzoic acid (DHBA) for reduction of the shikimate pool available for *p*-*HYDROXYCINNAMOYL-CoA SHIKIMATE* *HYDROXYCINNAMOYL TRANSFERASE* (*HCT*, Figures 1 and 3) in the cytosol (this is a crucial metabolic entry point for the synthesis of the most important lignin monomers, coniferyl and sinapyl alcohols). In vitro experiments have suggested that at least one poplar HCT can accept DHBA as a co-substrate instead of shikimate to produce *p*-coumaroyl-DHB (Eudes et al. 2016a). Although the DHB-tolerance of subsequent enzyme reactions in the PPP remains to be proven, two-dimensional nuclear magnetic resonance (NMR) analysis suggests that DHB may be compatible with the radical coupling reactions that assemble polymeric lignin in plants. This results in pendent DHB moieties (ester-linked groups) in lignin as well as backbone-integrated DHB units, ultimately producing a novel type of ‘Zip-lignin’ by using ML-DHB conjugates (Unda et al. 2022).

The *QsuB* gene has been expressed in the bioenergy crop switchgrass using the stem-specific promoter of an *O-METHYLTRANSFERASE* gene (*pShOMT*) from sugarcane (Hao et al. 2021). Under the controlled greenhouse condition, engineered switchgrass lines expressing a *pShOMT*::*QsuB* construct showed reductions in lignin content (by 12–20%), improvement in biomass saccharification efficiency (21–30%), and accumulation of higher amounts of DHB (2- to 3-fold) compared with the control plants. In addition, all transgenic lines showed no noticeable phenotype nor growth defect and were visually indistinguishable from each other or compared with WT plants (Hao et al. 2021). Later, three transgenic lines containing a *pShOMT*::*QsuB* construct were tested in the field for three growing seasons (Eudes et al. 2023), resulting in the improvement of dry biomass yields in two lines (11% and 16% increase each), slight increase in saccharification efficiency (average 5.0%), and no changes in lignin contents compared with those in the WT (Eudes et al. 2023). Analysis of lignin composition also revealed a minor but significant increase in the S/G ratio in the *QsuB*-expressing lines. This study demonstrated good performance of engineered plants in the field but showed that the cell wall changes in the greenhouse were not completely replicated in the field, emphasizing the need to validate engineered plants under relevant field environments (Eudes et al. 2023).

#### e) Reduction in the degree of lignin polymerization with C_6_C_1_ monomers

Another microbial approach for the overproduction of rare alternative monomers is to reduce the lignin polymerization degree (DP). Biosynthesis of the ‘DP reducers’ was achieved by expressing a *HYDROXYCINNAMOYL-*CoA *HYDRATASE-LYASE* (*HCHL*, Figure 3) from the bacterium *Pseudomonas fluorescens* in higher plants (Eudes et al. 2012; Mayer et al. 2001) and from *Rhodopseudomonas palustris* (*couA*; Sakamoto et al. 2020). HCHL respectively converts feruloyl-CoA, *p*-coumaroyl-CoA, and caffeoyl-CoA into equimolar quantities of different C_6_C_1_ monomers (vanillin, 4-hydroxybenzaldehyde, and 3,4-dihydroxybenzaldehyde) with acetyl-CoA (Mayer et al. 2001). This results in an increase in number of C_6_C_1_ end-groups in lignin. Compared with canonical C_6_C_3_ MLs (H, G, and S), C_6_C_1_ monomers may reduce the polymerization properties because they lack a propanoid side-chain and its conjugated double bond, preventing condensation at the β-position. This approach is intended to increase the amount of C_6_C_1_ monomers in lignin to reduce its DP and to be easily transferable from model plant species to crops as it does not require a particular genetic background. In Arabidopsis, *HCHL* was expressed under the control of the SCW cellulose synthase (*CesA4*) promoter to specifically target lignifying tissues and mitigate adverse effects. Several *HCHL*-expressing Arabidopsis lines showed no growth defects nor a biomass yield penalty, but 15–77% increases in saccharification efficiency after conventional pretreatment (hot water, dilute alkali, or acid), despite having a lignin content similar to that of the WT controls. Furthermore, analysis of the lignin monomeric composition revealed the presence of various C_6_C_1_ aromatics (Eudes et al. 2012). The size-exclusion chromatography (SEC) elution profiles of the dissolved cellulolytic enzyme lignin differed between WT and *ProCesA4:HCHL* plants. In particular, the SEC fractions corresponding to the smallest lignin fragments was increased by 55% in transgenic lines, demonstrating that the DP of lignin purified from *ProCesA4:HCHL* plants was reduced compared with that of the WT (Eudes et al. 2012). A similar study using the same construct expressed in transgenic Arabidopsis revealed that the DP of lignin from the WT and *HCHL* line was marginally different, whereas the biomass from the *HCHL* line containing C_6_C_1_ monomers showed an increase in pretreatment efficiency with deep eutectic solvents (DES) and released up to 34% more fermentable sugars compared with the WT biomass (Kim et al. 2019).

Augmenting the amounts of another C_6_C_1_ monomer, *p*HBA, in lignin represents an opportunity to add value to bioenergy crops similarly to *p*CA (Lin and Eudes 2020). By expressing the bacterial *CHORISMATE PYRUVATE LYASE* (*ubiC*, Figure 3) gene in sorghum, *p*HBA was incorporated into lignin, but the incorporation of this monomer did not significantly decrease the DP of lignin compared with that of the WT (Wang et al. 2021), in a similar manner to a recent report for transgenic *ubiC* poplar (Mottiar et al. 2023a). Nevertheless, the Glc released from the biomass of transgenic sorghum was as much as 12% higher than that from the WT biomass after pretreatment with one type of natural DES that was synthesized from choline chloride and *p*HBA (Wang et al. 2021). In addition, 10% more Glc and 50% more xylose (Xyl) were obtained following alkaline pretreatment from transgenic poplar (Mottiar et al. 2023a). The roles of the lignin C_6_C_1_ monomer in the decrease in lignin DP may require clarification.

### (4) Modulation of lignin polymerization using de novo designed enzymes

Lignin polymerization begins with the dehydrogenation (single-electron oxidation) of MLs that produce phenoxy radicals. Dehydrogenation may be initiated by oxidative enzymes (peroxidases/laccases, Figures 1 and 3) at the *p*-hydroxyl (4-OH) site of the aromatic ring and is followed by electron resonance to generate free radical intermediates (Bhuiya and Liu 2010). Subsequent coupling of the phenoxy radicals to each other, or to the growing polymer during lignification, forms the lignin polymer. Therefore, *p*-hydroxyls of phenolics are critically important for oxidative coupling of phenoxy radicals to form polymers. Creation of an enzyme to substitute the *p*-hydroxyl of MLs with a methyl moiety might interfere with lignin synthesis. Generally, *O*-methyl transferases (OMTs) are suitable for this purpose because these enzymes are often strictly regiospecific but are also capable of converting a broad range of (aromatic) substrates (Dippe et al. 2022). A natural evolution and selection approach resulted in the creation of several specialized OMT series that catalyze specific chemical reactions. As a target, *Clarkia breweri* (ISO) EUGENOL 4-OMT (CbIEMT) was selected and the phylogeny of plant phenolic OMT pointed to the existence of a batch of evolutionarily “plastic” amino acid residues in the active site. Next, adopting a path of directed evolution of one amino acid substitution at a time, and using a structure-based iterative site-saturation mutagenesis strategy, a novel *ML 4-OMT* enzyme designated “*MOMT3*” was created from *CbIEMT* after 96-well plate-based and radioactive enzyme assay screening (Bhuiya and Liu 2010). *MOMT4* (Figure 3), which bears one additional amino acid substitution (H169F) in the active site of MOMT3, a triple mutant variant of *CbIEMT* (T133L/E165I/F175I), was expressed in Arabidopsis and poplar under the control of the *Phaseolus vulgaris PHENYLALANINE AMMONIUM LYASE-2* (*PAL2*) promoter, resulting in the formation of 4-*O*-methyl coniferyl and sinapyl alcohols lacking an aromatic hydroxyl group, which were unable to be incorporated into growing lignin polymers. The transgenic plants exhibited lower lignin contents (as much as 24% reduction in Arabidopsis, and a modest reduction in poplar), a higher saccharification efficiency (up to 22% increase in Arabidopsis and 62% increase in poplar), but no significant growth penalty (Cai et al. 2016; Zhang et al. 2012). Interestingly, *MOMT4* expression in Arabidopsis resulted in the reduction of both G- and S-lignin subunits without alteration in the S/G ratio (Zhang et al. 2012), whereas in poplar S-lignin accumulation was drastically reduced without impairing the G-lignin subunits and, consequently, significantly reversed the S/G ratio of poplar lignin (Cai et al. 2016). Although *MOMT4* displays a discernible, yet less dominant, substrate preference for sinapyl alcohol, when it is expressed in the G unit-rich Arabidopsis, its kinetic effect may be masked or compromised. When *MOMT4* acts in an environment in which the S-monomeric substrate is more prominently available, such as poplar, its kinetic propensities may result in its dominantly modifying sinapyl alcohol, thus triggering a metabolic response entailing a more severe disruption of S-lignin formation (Cai et al. 2016).

To address the undesirable substrate preference of MOMT4, the authors assumed that specific remodeling of the active site of a ML 4-OMT would create an enzyme that specifically methylates coniferyl alcohol. This hypothesis was tested by using structure-guided combinatorial saturation mutagenesis to redesign the binding pocket of a substrate-promiscuous MOMT5, which is a penta-mutant variant carrying two additional substituted amino acids (F166W and H169F) from MOMT3, in a stepwise manner. A functionally specialized variant, MOMT9 (Figure 3), was generated that harbored four additional substitutions (M26H/S30R/V33S/T319M) from MOMT5. MOMT9 shows substantially altered substrate preference for coniferyl alcohol. Crystallization and structural determination revealed that MOMT9 has a significantly smaller substrate binding pocket compared with the parent enzyme, which impedes binding of the bulkier sinapyl alcohol and explaining its strong specificity for coniferyl alcohol (Cai et al. 2015).

Recently, it has been demonstrated that *OsC4H* promoter-driven expression of *MOMT4* and *9* in rice resulted in differential but drastic suppression of lignin deposition, resulting in more than 50% decrease in G-lignin content and as much as 90% reduction in S-lignin content in transgenic lines (Dwivedi et al. 2024). Moreover, the contents of AX-bound FA were reduced by up to 50% and the amounts of tricin in the lignin fraction were substantially reduced. In particular, the drastic reduction in AX-bound FA was expected to result in a decrease in crosslinking between AX and lignin polymer chains, contributing to an improvement in enzymatic cell wall digestibility. Accordingly, the cell wall structural and compositional changes resulted in as much as a 30% increase in the saccharification yield of the destarched rice straw biomass after diluted acid pretreatment (Dwivedi et al. 2024). However, dwarfism in asexually propagated transgenic offspring of MOMT lines has been observed, probably because of the toxic build-up of phenolic substances or accumulation of detrimental effects in successive generations. In addition to dwarfism, the *MOMT4*/*9* transgenic lines failed to develop viable pollen grains and consequently were sterile. The expression of *MOMT4*/*9* driven by the *OsC4H* promoter not only suppressed lignin formation but also substantially reduced cell wall FA and *p*CA deposition. In addition, *OsC4H* was highly expressed in the tapetum layer of the anther and was involved in exine formation (Dwivedi et al. 2024).

### (5) Stacking reduced lignin recalcitrance with value-added green chemicals

A recent detailed technoeconomic analysis has identified economically advantaged platforms and the break-even point for plants and microbes for four exemplar compounds (*p*HBA, catechol, muconic acid, and 2-pyrone-4,6-dicarboxylic acid [PDC]) (Yang M et al. 2022). The results suggest that accumulation rates exceeding approximately 1.0% dry weight (DW) in plants are likely to outcompete microbial production of bioproducts based on the assumption that the remaining biomass can be valorized through conversion to a liquid fuel.

**Figure 4:**
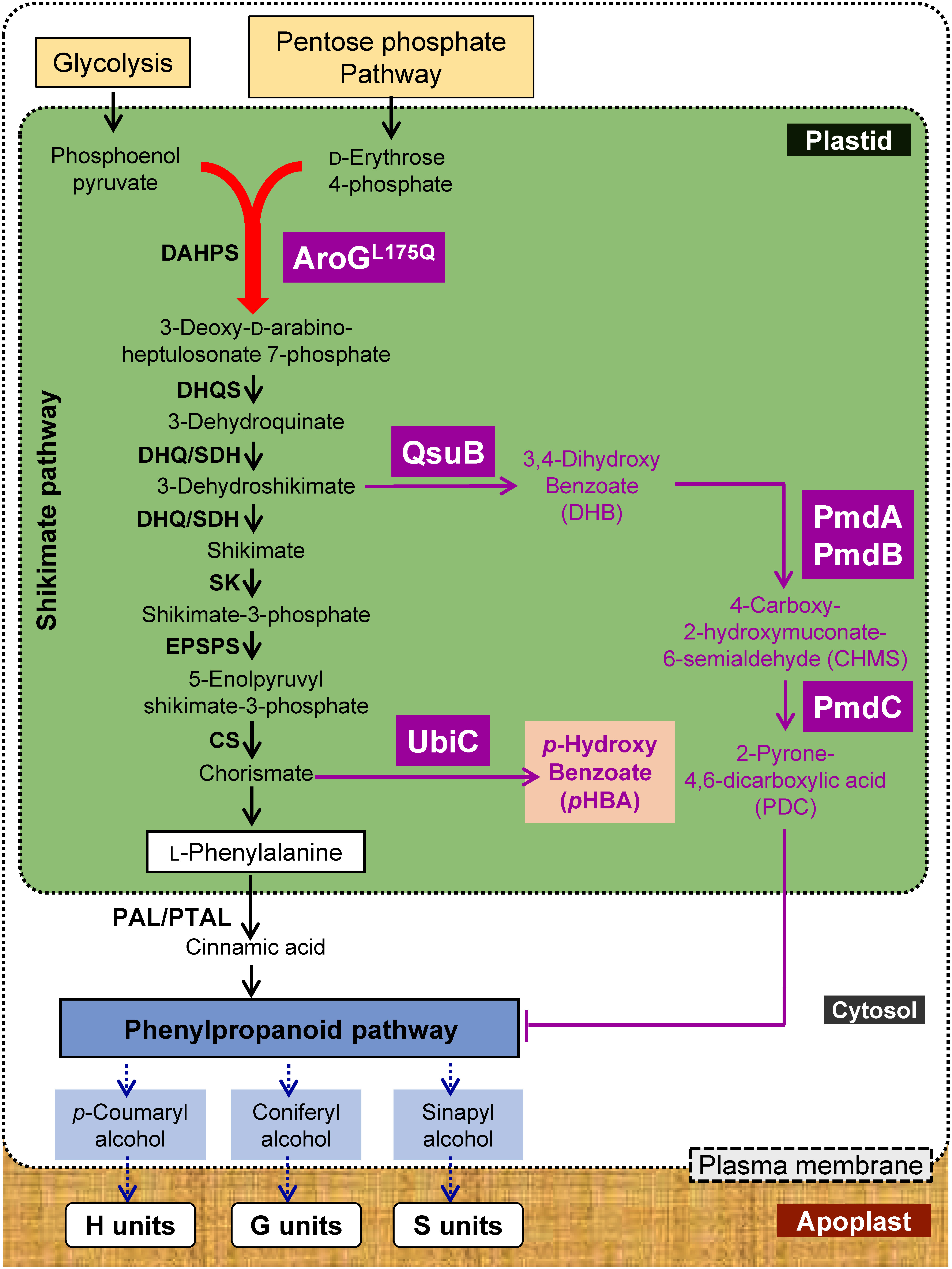
Figure 4. Engineering strategies for 2-pyrone-4,6-dicarboxylic acid and *p*-hydroxybenzoate overproductions in plants. Schematic diagram of the shikimate, aromatic amino acid metabolic networks in plants (Adapted from Yokoyama et al. 2022) and engineering approach for PDC and *p*HBA. Arrows represent a single enzymatic step (See also Figure 1). Enzymes in magenta indicate introduction of foreign genes. AroGL175Q, 3-deoxy-d-arabino-2-heptulosonate 7-phosphate synthase with L175Q mutation; *QsuB*, 3-dehydroshikimate dehydratase; *PmdA*, 3,4-dihydroxybenzoate (DHB) 4,5-dioxygenase α-subunit; *PmdB*, DHB 4,5-dioxygenase β-subunit; *PmdC*, 4-carboxy-2-hydroxymuconate-6-semialdehyde dehydrogenase.

*p*HBA can serve as a C_6_C_1_-precursor for the manufacture of nutraceuticals, cosmetic ingredients, drugs, fibers, platform chemicals, and DES (Wang et al. 2018). Several studies have demonstrated the expression of *ubiC* in plants to convert the plastidial chorismate from the shikimate pathway into *p*HBA (Lin et al. 2022; Mottiar et al. 2023a; Wang et al. 2021) (Figure 4). The potential bioenergy crop sorghum was recently transformed to express *ubiC* under the control of the rice ubiquitin promoter (*pRubi2*) co-expressed with plastid-targeted feedback-resistant *3-DEOXY-D-ARABINO-HEPTULONATE-7-PHOSPHATE SYNTHASE* (*DAHPS*, *AroG^L175Q^*, Figure 4) from *Escherichia coli* and driven by the maize cellulose synthase promoter (*pZmCesA10*) to increase carbon flux through the shikimate pathway and enable overproduction of *p*HBA in planta (Lin et al. 2022). In the field, when harvested before grain maturity, transgenic lines had *p*HBA contents of 0.8% and 1.2% DW (Yang M et al. 2022). Only a slight reduction (11–15%) in biomass yield was observed in transgenic plants grown under a natural environment.

Two-dimensional NMR analysis has revealed the presence of 4-hydroxybenzoate ester groups on lignin isolated from *ubiC*-engineered sorghum. As a result, the amount of Glc released from the pretreated transgenic biomass was as much as 12% higher than that from the pretreated WT biomass after pretreatment with DES (Wang et al. 2021). However, in a separate study (Lin et al. 2022), the biomass from the same engineered lines of sorghum released a similar amount of Glc to that of the WT controls, but Xyl yields were reduced by 6.7–8.5% after pretreatment with the ionic liquid cholinium phosphate. This finding implies that screening of pretreatment methods is important for *ubiC*-engineered biomass.

PDC is a promising building-block chemical for the manufacture of performance-advantaged polymers (Shikinaka et al. 2018). Engineered *E. coli* and *Pseudomonas putida* strains containing genes from *Sphingobium* sp. SYK-6 or *Comamonas testosteroni* encoding *DHB* *4,5-DIOXYGENASE* (*PmdA* and *PmdB*, Figure 4) and *4-CARBOXY-2-HYDROXYMUCONATE-6-SEMIALDEHYDE (CHMS) DEHYDROGENASE* (*PmdC*, Figure 4) enable the conversion of DHB into PDC. In this pathway, CHMS produced from DHB by PmdA and PmdB is non-enzymatically converted to its intramolecular hemiacetal form before conversion into PDC by PmdC (Lin et al. 2022). Recently, gene stacking that enables two-step conversion of DHB, the product of *QsuB*, into PDC has been proposed (Lin et al. 2022). In tobacco leaves, transient expression of bacterial feedback-resistant *AroG^L175Q^* and *QsuB* produced high titers of DHB, which was, in turn, efficiently converted into PDC upon co-expression of *PmdA*, *PmdB* and *PmdC*. In Arabidopsis, five genes (*AroG^L175Q^*, *QsuB*, *PmdA*, *PmdB*, and *PmdC*) were stacked on a single construct with five xylem-specific promoters in WT plants, resulting in PDC titers of approximately 3% DW in plant biomass. Moreover, all PDC-producing lines showed strong reduction in lignin content of stems (by 30–45%) and improvement in biomass saccharification efficiency (increases of 37–77% for Glc and 46–84% for Xyl). Importantly, most transgenic lines showed no reduction in biomass yields (Lin et al. 2021b). Therefore, it has been concluded that engineering plants with the proposed de novo PDC pathway provides an avenue to enrich biomass with a value-added co-product, while simultaneously improving biomass quality for the supply of fermentable sugars.

## Three strategies for redesigning the cell wall glycan synthetic pathway

In contrast to the relatively sophisticated strategies for lignin manipulation, the biotechnological potential of cell wall glycan synthesis (CWGS) manipulation that has been tested in model plants remains uncertain. A major bottleneck in redesigning CWGS is GTs (Voiniciuc 2023). For in vitro studies, the chemical synthesis of oligosaccharides, glycan arrays, and direct imaging of single glycan molecules facilitates high-throughput screening of GTs to determine their molecular function (Anggara et al. 2023; Ruprecht et al. 2020). However, many plant GTs, particularly those with multiple transmembrane spans, such as *CELLULOSE SYNTHASE A* (*CESA*) (GT2) and *CALLOSE SYNTHASE* (*CalS*) (GT48), are challenging to purify in active form (Voiniciuc 2023). These current limitations restrict most studies to performing basic biochemical analyses, such as the measurement of in vitro activities of GTs, rather than assessment of biotechnological applications. Nevertheless, there are at least three strategies to manipulate CWGS, based on recent advances in cell wall engineering, as follows.

### (1) Incorporation of kinks from β-1,3-glucan linkages into cellulose macrofibrils

The intussusception of callose (linear β-1,3-glucan) into macrofibrillar cellulose has been achieved in poplar by expression of a gain-of-function mutated CalS gene under the control of a xylem-specific promoter, which resulted in improvement in cellulose saccharification efficiency (Bourdon et al. 2023). Multiscale analysis of the transgenic poplar, including solid-state NMR experiments, has shown that ectopic deposition of callose modulates cell wall porosity, water absorption, lignin content, and increases the lignin–cellulose distance, ultimately resulting in a substantial decrease in biomass recalcitrance without a growth penalty. Sugar release in the constitutive lines was enhanced by as much as 37% and 55% for Glc and Xyl, respectively., Improvement in saccharification efficiency was even greater for the inducible lines, with monosaccharide release elevated by up to 81% for Glc and 94% for Xyl. Integration of ectopic polymers in biomass gives rise to novel physicochemical properties and presents new avenues for LC engineering (Bourdon et al. 2023).

### (2) Manipulation of the hexose (C6)/pentose (C5) ratio in matrix glycan assembly

Xylan is the most abundant structural glycan in plants after cellulose, comprising as much as 30% of the dry weight of the SCW in some dicotyledons and up to half of the SCW in grasses (Scheller and Ulvskov 2010). Xylan and lignin embed cellulose microfibrils in a tight matrix, thus restricting access of cell wall-degrading enzymes to the glycans (Lee et al. 2009). In addition, xylan is composed almost entirely of pentose sugars, which cannot be efficiently fermented (Young et al. 2010). Therefore, plants that have reduced amounts of xylan in the SCW, while still maintaining normal growth and development, would represent a valuable feedstock for biofuel production (Petersen et al. 2012). Multiple GTs participate in xylan biosynthesis in Arabidopsis. The xylan-deficient mutants *irx7* (GT47), *irx8* (GT8), and *irx9* (GT43) exhibit severe dwarfism and collapsed xylem (Brown et al. 2005). To repair the defective vessel systems, xylan engineering was undertaken to re-introduce xylan biosynthesis, specifically in the xylem vessels in the *irx7*, *irx8*, and *irx9* mutant backgrounds, by driving the expression of the respective GTs with the vessel-specific promoters of the *VASCULAR-RELATED NAC-DOMAIN PROTEIN VND6* and *VND7* transcription factors (Petersen et al. 2012). The best results were obtained by transforming the *irx7* mutant with the *pVND7:IRX7* construct. The transformants showed up to 23% reduction in Xyl content and 18% reduction in lignin content compared with the WT plants, while exhibiting WT growth patterns and morphology, as well as normal xylem vessels. In addition, the plants showed a 42% increase in saccharification yield after hot-water pretreatment. The xylan engineering system developed in Arabidopsis has potential for transfer to other biofuel crops, such as poplar, which has functional orthologs of the Arabidopsis *IRX* genes (Petersen et al. 2012). However, there is an increasing number of reports on pure xylan crystals and nanocrystals that showcase a wide range of potential utilizations for emulsification, carriers in drug delivery systems, environmental remediation, and dispersion agents (Johnson et al. 2023).

An additional approach to increase the C6/C5 ratio in LC is to increase the proportion of C6-rich glycans in SCWs, such as β-1,4-galactan (Liwanag et al. 2012) but not SCW type of cellulose (Brandon and Scheller 2020). The former non-cellulosic polymer is entirely composed of galactose residues (C6) and exists as side chains of rhamnogalacturonan I and relatively abundant in the SCW (Liwanag et al. 2012). Tension wood of aspen is reported to contain 10% β-1,4-galactan, which is hypothesized to induce gel-like properties, conferring the contractile driving force of tension wood as gelatinous fibers (Gorshkova et al. 2015). Liwanag et al. (2012) constitutively overexpressed *GALACTAN SYNTHASE 1* (*GALS1*, GT92), which is responsible for galactan biosynthesis, to achieve an increase of 50% galactan in the leaf cell wall in Arabidopsis. The authors boosted this further by co-expressing the *UDP-GLUCOSE/UDP-GALACTOSE-4-EPIMERASE 2* (*UGE2*) gene responsible for synthesizing the substrate of GALS1, UDP-galactose (UDP-Gal) (Gondolf et al. 2014). Finally, a gene stacking approach using yeast assembly (termed the jStack method) combined *GALS1* and *UGE2* with *UDP-RHAMNOSE/UDP-GALACTOSE TRANSPORTER 1* (*URGT1*), which transports UDP-Gal into the Golgi lumen for utilization by *GALS1*, resulting in a 50% increase in galactan content in the stem cell walls in Arabidopsis (Aznar et al. 2018).

As a powerful demonstration of plant synthetic biology, first, increase in galactan content and decrease in lignin content were combined by overexpressing *QsuB* (Unda et al. 2022) together with *GALS1*, *UGE2*, and *URGT1*. Second, increase in cell wall thickness was combined with the preceding manipulation through overexpression of *NAC SECONDARY CELL WALL THICKENING-PROMOTING FACTOR* *1* (*NST1*) to create an artificial-positive feedback loop (APFL) that increased the SCW density in Arabidopsis interfascicular fiber cells (Yang et al. 2013). Third, all of these modifications have been combined with decrease in xylan content by overexpressing the above-mentioned gene sets in a vessel-complemented xylan-deficient background (Petersen et al. 2012). Unfortunately, although the APFL system did not function well in this trial, the use of cell-type-specific promoters (*pC4H*, *pIRX5*, *pIRX8*, and *pCESA7*) avoided negative growth effects while generating plants that showed an approximately three-fold increase in the C6/C5 ratio and an increase in the release (by up to 73%) of fermentable sugars from the biomass (Aznar et al. 2018).

A series of multi-dimensional solid-state NMR experiments have been conducted to explore the effects of modulation of the galactan chain length on the SCW architecture in the transgenic lines generated by expressing *GALS1*, *UGE2*, and *URGT1* under the control of the SCW-specific *pC4H*, *pIRX5*, and *pIRX8* promoters, respectively (Gao et al. 2023). In the engineered plants, galactan closely associates with cellulose, and the elongated galactan chains block binding sites of cellulose for xyloglucan and the pectic backbone, both of which are minor SCW components (Scheller and Ulvskov 2010; Xiao and Anderson 2013). This results in a loosened SCW architecture, which promotes decomposition of the biomass.

### (3) Molecular docking of inorganic and organic systems to reduce LC recalcitrance

The recent development of iron-co-catalytic enhancement of dilute acid (DA) pretreatment of biomass is a promising approach for enhancing sugar release from recalcitrant LC. Compared with DA-alone pretreatment, the DA/iron co-catalyst pretreatment (followed by enzymatic hydrolysis) was more efficient in releasing sugars from filer paper (FP) and cotton linter (CL), showing enhancement of 16% for FP and 14% for CL compared with the Glc yield from DA-alone pretreatment (Wei et al. 2011). However, pretreatments in which the metal catalysts are directly added to biomass will increase unit operations and costs, and the high concentrations of metals required to penetrate the cell walls may induce potential environmental problems relating to water usage and disposal (Yang et al. 2016).

To overcome the limitation in ion diffusion properties in LC against exogenously applied iron ions during the pretreatment process, an alternative approach that overexpresses soybean ferritin intracellularly (*FerIN*) or extracellularly (*FerEX*) by adding a signal peptide resulted in iron accumulation in transgenic plants under both normal and iron-augmented watering conditions in Arabidopsis (Lin et al. 2016). The resulting transgenic lines (*FerIN* and *FerEX*) both showed enhanced enzymatic saccharification efficiency after hot-water treatment without exogenous iron application; increases in the release of Glc (by 21% in *FerEX* and 17% in *FerIN*) and Xyl (by 34% in *FerEX* and 16% in *FerIN*) were observed from the biomass compared with the findings from the empty vector (EV) lines (Lin et al. 2016). The inflorescence biomass from both transgenic lines was respectively increased (by 18% in *FerEX* and 11% in *FerIN*) compared with that of the EV lines (Lin et al. 2016; Wei et al. 2015).

To target iron-binding proteins (IBPs) that are in closer proximity to cell wall glycans after their secretion, CBM-IBP fusion polypeptides have been engineered by docking CBM11 from *Clostridium thermocellum*, which binds primarily to β-1,4-glucan and grass-specific β-(1,3;1,4)-glucan, and the synthetic blood IBP after multi-step screening of five CBM species and two IBPs (Yang et al. 2016). The *CBM-IBP*-transformed Arabidopsis and rice plants showed significant increases in iron accumulation (by 60–70% in Arabidopsis and approximately 60% in rice) and biomass conversion after hot-water treatment in the absence of exogenously applied iron (20% and 25% increases in Glc in Arabidopsis and rice, respectively, and 15% increase in Xyl in both species) compared with the respective controls (Yang et al. 2016). Metal-hyperaccumulating plants often show reduced growth, whereas the Arabidopsis transgenic lines showed no detrimental effects on plant growth. The shoot height of *CBM-IBP*-transformed rice lines was reduced compared with that of the WT, whereas the former plants developed a greater number of tillers to offset the shorter stature. As a result, the dry weight and number of seeds of *CBM-IBP-*transformed rice were greater than those of the WT under the greenhouse condition (approximately 40% more grains per *CBM-IBP*-transformed plant compared with the findings for the WT rice; Yang et al. 2016).

Recently, this technology has been applied in switchgrass by stacking of the intracellular soybean ferritin (*FerIN*) and an improved version of cell wall-bound *CBM-IBP* having four tandem repeats of the IBP peptide (*IBPex*) (Lin et al. 2021a). The *FerIN*-expressing switchgrass showed a 15% increase in plant height and 65% higher biomass yield, whereas the stacked *FerIN*/*IBPex* transformants showed increases of up to 30% in plant height and 115% in biomass yield. The *FerIN* and *FerIN*/*IBPex* switchgrass respectively had 27% and 51% higher in planta iron accumulation than the EV control. The biomass of the engineered switchgrass plants, especially the *FerIN*/*IBPex* transformants, showed reduced recalcitrance and improved enzymatic saccharification efficiency after hot-water treatment without additional harsh chemicals or exogenous iron supplement (approximate 14% increase in Glc release in *FerIN* lines and up to 24% increase in Glc release in *FerIN*/*IBPex* lines, but no significant effects on Xyl release were observed in both lines). These results demonstrated that iron incorporation can be applied as a universal approach to reduce barriers to thermochemical conversion and facilitate plant biomass deconstruction, even for highly recalcitrant species such as switchgrass (Lin et al. 2021a).

## Conclusion and perspective

Understanding how genes control higher-order architectural features of LC, and how those features then impact on biomass conversion processes, would facilitate catalyst design and reduce reactor residence times and energy inputs for the mechanical breakdown of LC particles (Carpita and McCann 2020). Recent advances in understanding LC biosynthesis and its regulation have enabled partial reduction in the LC recalcitrance in various model plants. Engineering approaches not only include alteration of the expression of genes that participate in LC synthesis, but also the redesign of conventional enzymes to confer novel substrate specificity, conversion of cofactors and metabolic intermediates, and combination with inorganic chemistry. Several strategies demonstrated in model plants have been gradually transferred to crop species. To date, low-lignin alfalfa with *CCoAOMT* knockdown by RNAi silencing technology for improved forage quality (branded as HarvXtra®) represents the only commercialized LC-modified crop (Barros et al. 2019), while crops subjected to LC engineering via synthetic biology, including CRISPR-Cas9 gene editing, have not yet reached the market (Liu and Eudes 2022). Several conditions must be met for further advances, such as enhanced transformation efficiency of commercial species or genotypes, capacity for large-scale transformation experiments, as well as (crucially) field trials to confirm greenhouse phenotypes at the harvest stage (Myburg et al. 2019).

The adoption of plant production systems by industry is hindered by the fact that mammalian and other cell cultures are much more well-established and better characterized in an industrial setting, making it difficult for plant-based processes to gain a foothold in the market. Therefore, additional advantages of plant-based systems may be essential to tip the balance in favor of sustainable plant-derived products, as discussed by Buyel (2018). In this respect, designing mid- to high-value co-products that can be derived cost-effectively from the residual LC, as described in this review, may be crucial for an additional increase in revenue. One interesting future research direction is integration of the several steps of molecular pharming for recombinant proteins and green chemicals in an enclosed plant-based production facility, such as a vertical farm, in combination with LC biorefineries after the breeding of the engineered plants with the essential help from synthetic biology. It is an exciting time for plant bioengineers addressing the grand challenge of LC manipulation to achieve the final goal.
